# Combined Effects of Postactivation Performance Enhancement and Caffeine Intake on Explosive and Anaerobic Power in Recreationally Active Males

**DOI:** 10.1016/j.cdnut.2025.107437

**Published:** 2025-04-05

**Authors:** Nazila Heydari, Mahsa Shojaee, Javad Nemati, Ahmad Reza Dehghani, Alireza Niknam, Fereshte Eftekhari, Mohammad Hemmatinafar

**Affiliations:** Department of Exercise Physiology, Faculty of Education and Psychology, Shiraz University, Shiraz, Iran

**Keywords:** post-activation performance enhancement (PAPE), caffeine supplementation, explosive power, anaerobic capacity, sprint performance

## Abstract

**Background:**

Both post-activation performance enhancement (PAPE) and caffeine (CAF) are known to acutely improve physical performance. However, their combined effects on multiple performance outcomes in recreationally active individuals remain underexplored.

**Objective:**

This study explores the combined effects of PAPE and caffeine CAF supplementation on explosive power, sprint performance, and anaerobic capacity in recreationally active men.

**Methods:**

In a double-blind, crossover design, 20 participants completed 4 sessions with distinct interventions: placebo (PLA) with usual warm-up (No-PAPE + PLA), PAPE + PLA, CAF without PAPE (No-PAPE + CAF), and PAPE + CAF. After CAF (6 mg CAF/kg body mass) or PLA ingestion, participants performed warm-ups. They underwent physical tests, including vertical jump height (VJH), standing long jump (SLJ), 40-yard dash, and the running-based anaerobic sprint test (RAST). Data were analyzed using 1- and 2-way repeated measures analysis of variance and Bonferroni post hoc tests (*P* < 0.05 considered significant).

**Results:**

The PAPE + CAF condition yielded significant improvements in VJH compared with other conditions (*P* < 0.01), although the 40-yard dash times improved significantly in No-PAPE + CAF, PAPE + PLA, and PAPE + CAF conditions compared with PLA (*P* < 0.001). VJH also showed significant gains in PAPE + CAF compared with PAPE + PLA and No-PAPE + CAF (*P* < 0.01). Additionally, PAPE + CAF, PAPE + PLA, and No-PAPE + CAF produced notable increases in RAST metrics, including peak power, average power, minimum power, total time, and anaerobic capacity compared with No-PAPE + PLA (*P* < 0.001), although fatigue index differences remained nonsignificant. No significant effects were found in SLJ (*P* > 0.05).

**Conclusion:**

These findings highlight a synergistic effect between PAPE and CAF in enhancing short-term explosive performance, offering practical strategies for optimizing high-intensity activities in recreationally active individuals.

## Introduction

Optimizing athletic performance, particularly in recreationally active individuals, requires enhancing explosive power [1,2]—the capacity to generate maximal force within a short time frame during high-intensity, all-out muscular efforts such as jumps or throws [3]—and anaerobic power [2,4], which reflects the body’s ability to rapidly produce energy through nonoxidative metabolic pathways. Although aerobic capacity has been the primary focus of many studies, anaerobic power and explosive strength are also critical components of athletic performance [2,4] and have received comparatively less attention in the literature. Many sports and physical activities, including those commonly practiced by recreationally active individuals, require the ability to generate high power outputs in short durations, relying on anaerobic energy pathways [2,4]. Enhancing these attributes can improve movement efficiency, speed, and overall performance in high-intensity efforts.

Among the approaches that have attracted increasing interest are postactivation performance enhancement (PAPE) [[5], [6], [7], [8], [9]] and caffeine (CAF) supplementation [[10], [11], [12], [13]]. Each has demonstrated independent benefits in enhancing various facets of physical performance [10,[14], [15], [16], [17]]. PAPE refers to a temporary increase in muscle force and power output following a high-intensity conditioning activity, typically resulting from neural and muscular adaptations prompted by the preceding stimulus [8,18]. CAF, a well-established ergogenic aid, acts by stimulating the central nervous system, lowering perceived exertion, and improving force production and endurance capacity [[12], [13], [14]]. Thus, examining the potential synergistic impact of combining PAPE with CAF intake on explosive and anaerobic power could substantially contribute to the current understanding of optimizing athletic performance [5,19].

The phenomenon of PAPE is based on the physiological premise that a muscle’s prior contraction can temporarily improve its subsequent performance [8,18]. Initially described as postactivation potentiation (PAP), this effect has been linked to both neural and muscular adaptations, such as increased phosphorylation of myosin regulatory light chains, enhanced muscle activation, and improved motor unit recruitment [8,9,18,20]. More recent studies have begun to distinguish between PAP and PAPE, with findings indicating that PAPE is more commonly associated with improvements in power-oriented movements relevant to sports, as opposed to isolated contractions [8]. Although PAP and PAPE are occasionally used interchangeably, PAPE specifically denotes performance gains following preparatory activities that closely replicate the power demands of the sport [8,17]. For example, Blazevich and Babault (2019) [8] reported that PAP is primarily observed in twitch contractions and electrically induced muscle activations. In contrast, PAPE has a more significant effect on voluntary, high-power actions such as jumping and sprinting [8]. Likewise, Seitz and Haff (2016) [21] identified that PAPE is more evident in athletes executing countermovement jumps (CMJs) and short sprints after heavy resistance exercises, highlighting its relevance in sports performance. However, recent findings by Fischer and Paternoster (2024) [22] challenge this perspective by demonstrating that although PAP elicited significant increases in peak torque and rate of torque development, no measurable PAPE effect was observed, regardless of whether the conditioning contraction was voluntary or electrically evoked. This absence of a detectable PAPE response at the designated assessment intervals raises questions about the assumed neural mechanisms underpinning the phenomenon and whether PAP directly contributes to PAPE [22]. The discrepancies between these findings underscore the need for further research to refine the conceptual distinction between PAP and PAPE, particularly in relation to their time-dependent effects and practical implications for athletic performance.

The mechanisms behind PAPE encompass elevated muscle temperature, intracellular fluid shifts, and increased neural activation, each contributing to temporary improvements in muscle function [8,9,19]. PAPE conditioning protocols typically involve high-intensity resistance exercises, such as squats or deadlifts, conducted before power-oriented tasks, such as vertical jumps or sprints [5,6,16,17,20]. Additionally, plyometric exercises have been utilized as an effective PAPE strategy [20,23], given their focus on explosive, rapid movements that engage the stretch-shortening cycle (SSC). Plyometrics, such as depth jumps, bounding, and hurdle hops, activate the neuromuscular system in a way that primes the muscles for subsequent high-power activities [15,24]. Studies have indicated that plyometric-based PAPE protocols can positively impact sprint and jump performance, although the effectiveness of these protocols depends on factors such as exercise selection, intensity, rest intervals, and the athlete’s conditioning level [15,21]. Both resistance and plyometric exercises serve as valuable components in PAPE protocols aimed at optimizing short-term gains in explosive performance [21,25].

Although researchers pay considerable attention to the influence of training techniques like PAPE on performance, the interaction between these techniques and synergistic nutritional strategies has also attracted interest [5,19]. A critical question is which nutritional interventions can augment the effectiveness of PAPE. The findings of this study align with prior research suggesting that CAF may exhibit synergistic effects with PAPE in improving athletic performance [7,19,23]. Specifically, Zhang et al. [7] demonstrated that CAF ingestion combined with PAPE led to enhanced peak performance compared with PAPE alone, highlighting the potential for CAF to amplify the neuromuscular benefits of PAPE. Similarly, Ouergui et al. [26] reported that CAF combined with PAPE improved agility and kick speed in female taekwondo athletes, further supporting the notion of CAF as an ergogenic aid in power-based activities. However, Filip-Stachnik et al. (2022) [5] found no significant synergistic effect on vertical jump performance in trained female volleyball players, suggesting that factors such as sex differences and training history may influence the outcomes of CAF-PAPE interactions. These mixed findings highlight the complexity of individual responses to PAPE and CAF, necessitating further research to identify the conditions under which their combination yields the most pronounced benefits. CAF is a central nervous system stimulant that enhances muscle performance by increasing alertness, reducing perceived effort, and improving neuromuscular function [10,[12], [13], [14],^19^]. It facilitates greater calcium (Ca^2+^) release from the sarcoplasmic reticulum, improving force production during high-intensity physical activities [12,13]. In addition, the antagonistic effect of CAF on adenosine receptors could also play a role in the ergogenic effect of CAF [10,12,26]. There is also evidence that acute CAF consumption can increase power performance such as jumping ability and anaerobic capacity [[10], [11], [12],^14^,^27^]. Although the precise mechanisms through which CAF enhances PAPE remain unclear, substantial evidence indicates that CAF can influence performance outcomes related to PAPE via multiple pathways [12,23,26,28]. These include neuromuscular stimulation, which enhances motor unit recruitment and firing rate and improves Ca^2+^ ion cycling within muscle fibers, which is crucial for muscle contraction efficiency [8,28]. Additionally, CAF may promote better interaction between muscle contractile proteins, optimize blood flow distribution to active muscles, and elevate muscle temperature, all of which are known to contribute to improved muscle performance [28]. Interestingly, the fundamental mechanisms underlying PAPE, as detailed by Blazevich and Babault (2019) [8], and the mechanisms of CAF action, as outlined by Lima-Silva et al. (2021) [28], exhibit considerable overlap in key physiological processes [8,28]. For example, PAPE and CAF are associated with enhanced calcium sensitivity and improved contractile force [8,28]. Furthermore, both processes are linked to alterations in muscle temperature and blood flow, which contribute to improved muscle function during high-intensity activities [8,28]. This overlap suggests that CAF may act synergistically with PAPE, potentially amplifying its effects on muscle performance by targeting these shared mechanisms [23,26]. However, some evidence has also shown that CAF consumption along with PAPE does not lead to higher performance [5]. The lack of adequate research on this topic and the discrepancies in the results suggest that additional studies are necessary in this field. In addition, it should be noted that CAF and PAPE can be combined in different protocols, doses, and methods, which may be effective due to their synergistic effect.

Despite substantial evidence supporting the individual benefits of PAPE and CAF supplementation, limited research has explored their combined effects on explosive and anaerobic power, particularly in recreationally active populations. Unlike trained athletes, recreationally active individuals may not possess the same neuromuscular adaptations necessary for traditional PAPE protocols, raising questions about their feasibility and effectiveness in this cohort. Therefore, this study aimed to investigate whether the combination of CAF intake and a PAPE protocol leads to greater improvements in explosive performance (e.g., VJH, sprint time) and anaerobic capacity (e.g., running-based anaerobic sprint test [RAST] parameters) compared with PAPE or CAF alone. We hypothesize that the concurrent use of PAPE and CAF will produce a synergistic effect, yielding superior performance enhancements than either intervention in isolation.

## Methods

### Participants

The study involved 20 male participants (age: 34.6 ± 7 y; body mass [BM]: 68.8 ± 5.6 kg; height: 176.5 ± 5 cm; BMI: 21 ± 2 kg/m^2^; training history: 5.8 ± 2.3 y), all of whom were recreationally active and had ∼2 y of consistent training experience (≥3 sessions per wk/combination of endurance and resistance exercises). Participants were required to meet specific inclusion criteria, which included engaging in regular exercise for the past 2 y, being physically healthy without any injuries, having no known CAF or coffee allergies, and having no smoking history. Participants were excluded if they experienced any infectious diseases (e.g., colds or flu) during the study, had significant musculoskeletal injuries, or used drugs or ergogenic supplements during the intervention period. Furthermore, the restriction on ergogenic supplements also applied to all forms of CAF supplements (such as capsules, tablets, powders, gels, or caffeinated beverages). Participants were only allowed to consume CAF/placebo (PLA) capsules under the conditions specified in the study design, and any consumption of CAF supplements outside the study protocol would result in their exclusion from the study. It is important to note that the consumption of CAF-containing food sources, such as tea, coffee, dark chocolate, and energy drinks, was only restricted 24 h prior to testing. Participants were allowed to consume these items according to their usual habits during the intervention, except for the 24-h period before the exercise test sessions. Before the intervention, the study procedures were thoroughly explained to the participants, and informed consent was obtained. The research received approval from the Ethics Committee of Shiraz University in Shiraz, Iran, and was conducted in accordance with the Declaration of Helsinki (ID: SEP/14033/48/4770). All data collection procedures were carried out at the School of Sport Sciences, Shiraz University, from 18 March, 2023 to 19 April, 2023.

### Sample size calculation and study design

The number of participants in this study was determined based on the study by Zhang et al. [7], according to which PAPE + CAF administration led to a significant improvement in anaerobic power compared with the control condition (effect size  =  0.58). Using G∗Power 3.1, considering a 95% confidence interval and analysis power of 0.80, it was determined that ≥19 participants were needed for this study. To ensure a sufficient sample size, 20 participants were selected for this study.

**FIGURE 1:**
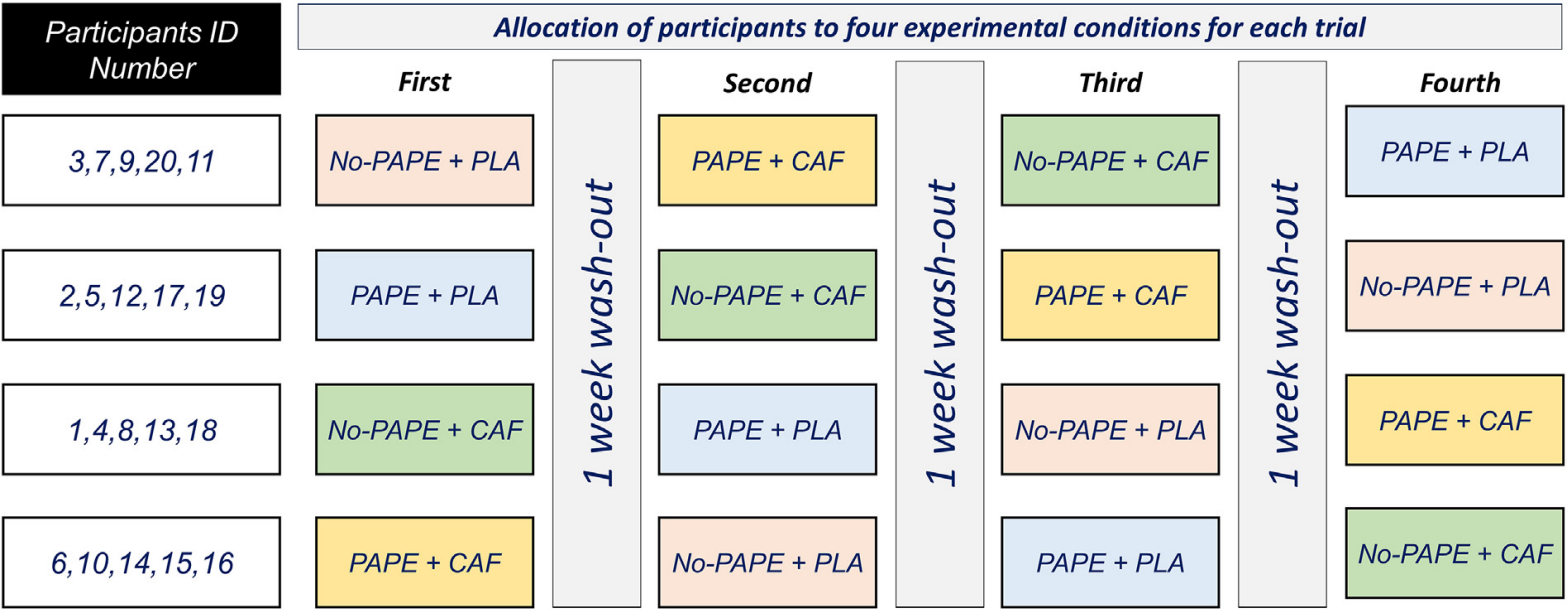
Study design and allocation of participants to 4 experimental conditions for each trial. CAF, caffeine; PAPE, postactivation performance enhancement; PLA, placebo.

**FIGURE 2:**
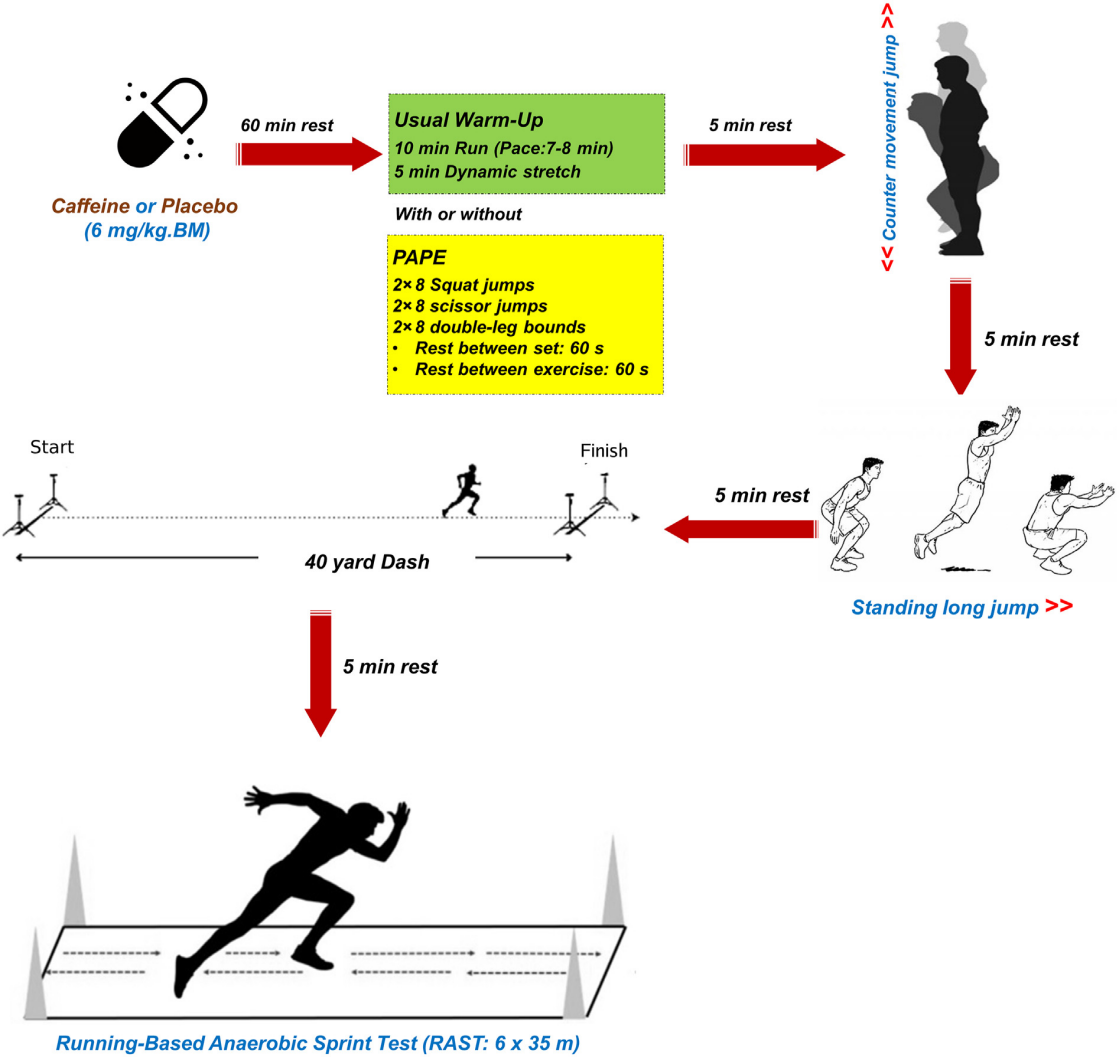
Supplementation and testing protocol session. BM, body mass; PAPE, postactivation performance enhancement.

This study employed a double-blind, placebo-controlled, crossover design to examine the combined effects of PAPE and CAF supplementation on explosive power and anaerobic capacity in recreationally active males. Before data collection commenced, participants attended an introductory session designed to familiarize them with the interventions and data collection methods. Each participant completed an informed written consent form and a health status questionnaire (Physical Activity Readiness Questionnaire) [29], which included specific inquiries about any previous CAF allergies. Participants were instructed to avoid consuming any food or beverages containing CAF for 24 h prior to the supplementation and exercise testing sessions. This restriction covered common CAF sources like coffee, tea, energy drinks, and chocolate. Subsequently, participants completed 4 distinct exercise testing sessions, evaluating lower-body explosive power, anaerobic capacity, and sprint performance. During these sessions, participants consumed either CAF (6 mg CAF/kg BM) or PLA (capsule containing starch), with a 1-wk interval between each testing session to allow for full washout of CAF effects (FIGURE 1, FIGURE 2). Participants were asked to verbally report any bothersome side effects experienced from supplement consumption to ensure that any adverse effects resulting from CAF intake did not negatively impact the final outcomes of the study. The double-blind design ensured that neither the participants nor the researchers knew which intervention (CAF or PLA) was administered during each session, minimizing bias and ensuring the reliability of the results. Identical capsules were used to ensure no visual distinction between the 2 preparations. The capsules were randomly assigned to participants in each testing session to prevent any pattern or predictability. Additionally, the researchers involved in data collection and analysis were also blinded to the type of supplement and conditions, further reducing the possibility of bias in interpreting results. In each of 4 experimental conditions, participants ingested either 6 mg CAF/kg BM or a placebo capsule 1 h before testing (Figure 2). This dose was selected based on safety and efficacy data in athletes [12,30]. The conditions included: *1*) placebo with usual warm-up only (No-PAPE + PLA, *n* = 5); *2*) PAPE warm-up with placebo (PAPE + PLA, *n* = 5); *3*) CAF without PAPE warm-up (No-PAPE + CAF, *n* = 5); and *4*) CAF with PAPE warm-up (PAPE + CAF, *n* = 5) (Figure 1). To minimize the influence of circadian variations, all testing was conducted at a consistent time aligning (between 10:00 and 11:00) with the athletes’ regular training schedules. All participants consumed the same breakfast containing 350 to 400 kcal (64% carbohydrates, 20% protein, and 16% fat) 2 h before the exercise test session. To control the impact of the pretest diet on athletic performance, participants were provided with a standardized dietary plan for the day before the test (calorie intake: 35 kcal/kg BM, 60%–65% carbohydrates, 15%–20% protein, and 20% fat) (Diet Organizer 3.1). Participants were instructed to avoid food and drink in the hour before testing and to avoid strenuous exercise 48 h before each trial.

### Warm-up and PAPE

After the random assignment to each condition, athletes ingested either PLA or CAF and then performed 1 of 2 warm-up protocols: *1*) a usual warm-up consisting of 10 min of running (pace 7–8 min, the time taken to run 1 km) followed by 5 min of dynamic stretching (No-PAPE); or *2*) a PAPE warm-up, which included the usual warm-up plus 2 sets of 8 repetitions of squat jumps (SJs), scissor jumps, and double-leg bounds with a 60-s rest between sets and exercises (PAPE) (Figure 2). Previous research has indicated that plyometric exercises can be an effective strategy for PAPE [15,31]. Plyometric exercise techniques were standardized by presenting 3 instructional videos to the participants before testing. In the SJs, participants performed a deep squat with feet shoulder-width apart before jumping explosively; in scissor jumps, they alternated leg positions midair; and in double-leg bounds, they executed forward jumps with maximal effort. A 5-min rest period postconditioning in PAPE conditions was included to maximize potentiation effects, as supported by previous findings. The 1-h postingestion period allowed for peak CAF plasma concentration, enhancing its ergogenic effects [12,30]. Each testing session was separated by ≥1 wk to ensure adequate recovery and CAF clearance.

### CAF supplementation

In the present study, CAF capsules were manufactured by the Omid Mokmmel Salamat Company (Nutrimed1958), approved by the Food and Drug Organization of Iran, at 6 mg CAF/kg BM. Identical capsules containing starch were used for the PLA group. CAF and PLA capsules were individually packaged and labeled to ensure blinding and proper dosing. Each participant received their assigned capsules in a sealed, coded package before each session. BM was measured using a calibrated digital scale (Seca 813) with a precision of ±0.1 kg. The recorded mass from the initial session was used to determine the CAF dose for all subsequent trials. At 60 min before each exercise session, participants randomly ingested PLA-containing capsules (starch) or CAF supplements (6 mg CAF/kg BM). The selected dosage and ingestion times were based on previous research that demonstrated the performance-enhancing advantages of low and moderate CAF doses [12,32]. It is noteworthy that participants’ CAF consumption was controlled, as they were instructed to avoid CAF sources for 24 h prior to the testing session.

### Exercise testing procedures

Participants engaged in various activities during each exercise test session, including vertical jumps, horizontal jumps, a 45-yard sprint, and the RAST. A 5-min rest period was implemented between each test to mitigate the residual fatigue resulting from each test (Figure 2).

#### Vertical jumping height

The vertical jumping height (VJH) was used to measure the vertical explosive power of the lower limbs. Vertical jumping height was assessed using a CMJ, recorded and analyzed via the Jump-Mat (Desi Kala) [33,34]. The Jump-Mat used in this study is equipped with a high-precision electronic timing system with accuracy of 0.001 s, ensuring precise measurement of flight time for VJH calculation. The validity of similar electronic switch mats (ESMs) for assessing jump height has been confirmed by Kenny et al. (2012) [35], demonstrating strong correlations with force plate measurements for CMJs (*r* = 0.996) and SJs (*r* = 0.958), with minimal absolute errors (0.01–0.02 m). Although ESMs may be less reliable for fast SSC movements such as drop jumps, they provide valid and reliable measurements for CMJs and SJs. Participants performed maximal-effort CMJs from a 90° knee and hip flexion depth with hands on hips to eliminate arm-swing influence. To ensure consistency, participants minimized knee flexion before landing and landed with both feet simultaneously. Each participant performed 3 attempts with 60 s of rest between jumps, and the best of these 3 jumps was used for statistical analysis. High test–retest reliability for the CMJ was confirmed with an intraclass correlation coefficient (ICC) of 0.96.

#### Standing long jump

The standing long jump (SLJ) was used to measure the horizontal explosive power of the lower limbs. Participants executed 3 maximal bilateral anterior jumps with an arm swing, and jump distance was recorded manually using a standard measuring tape from the starting line to the heel contact point on landing. The SLJ test has established validity and reliability [36]. Similar to the VJH, 3 attempts were recorded with 60 s of rest between each, and the best jump performance was used in the analysis [33].

#### 40-yard dash

The 40-yard dash was employed to evaluate sprinting speed [37]. Participants began from a stationary position, with 1 foot placed forward, holding this position for 2 s before initiating the sprint without any rocking motion. To improve performance, participants received coaching cues focusing on maintaining a low stance and executing strong arm and leg drives. They were instructed to run with maximum effort until they reached the finish line. Each participant was allowed 2 attempts, with the best time recorded to the nearest 2 decimal places. The timing started with the first movement and ended when the participant’s chest crossed the finish line, measured using a digital-photo electric timer (Desi Kala) with a precision of 1 ms. The validity of timing sensors in sprint time assessment has been demonstrated in studies such as Altmann et al. (2017) [38], which confirmed the accuracy of single-beam timing lights for measuring sprint times, with moderate to high ICCs for 30-m sprint times.

#### Running-based anaerobic sprint test

The RAST was used to evaluate anaerobic capacity, consisting of 6 maximal 35-m sprints with a 10-s rest between each, performed in alternating directions [39]. Sprint times were precisely recorded with a digital photoelectric timer (Desi Kala). Both time and power were recorded and analyzed. Sprint times (RAST-ST_1–6_) and total sprint time (RAST-TT) were directly measured using a digital photoelectric timer. Power was then calculated using these times along with participant BM and sprint distance, following established biomechanical equations ((1), (2), (3), (4), (5), (6)). The inclusion of power metrics allows for a more comprehensive analysis of anaerobic performance, as it accounts for both speed and force production over repeated sprints. Time-based metrics alone do not fully capture the energetic demands and fatigue patterns observed during the test. Peak power (RAST-PP) (Equation 2), the highest power output achieved during the sprints, and minimum power (RAST-MinP) (Equation 3), the lowest power output observed, both measured in watts (W). Average power (RAST-AvP) was calculated as the mean power output across all sprints (Equation 4), and the fatigue index (RAST-FI) quantified the decline in power, calculated by dividing the difference between RAST-PP and RAST-MinP by the total sprint time (Equation 5). Anaerobic capacity (RAST-AnC) was measured as the sum of all power outputs across the 6 sprints (Equation 6), providing a comprehensive assessment of anaerobic performance

Equation (1), (2), (3), (4), (5), (6):

(1) Power _watt_ = Body Mass _kg_ × Distance _meter_^2^ ÷ Time _seconds_^3^

(2) Peak Power (PP) _watt_ = the highest power measurement

(3) Minimum Power (MinP) _watt_ = the lowest power measurement

(4) Average Power (AvP) _watt_ = the sum of all 6 power values ÷ 6

(5) Fatigue Index (FI) _watt/s_ = (maximum power – minimum power) ÷ total time for the 6 sprints

(6) Anaerobic Capacity (AnC) _watt_ = Sum of all 6-sprint power

### Statistical analysis

All data were analyzed using descriptive and inferential statistical methods. Data distribution normality was determined using the Shapiro–Wilk test and were confirmed to be normally distributed (*P* > 0.05 for all variables). One-way and 2-way (4 × 6) repeated measures analysis of variance (ANOVA) was used to determine the main effect on performance. Bonferroni post hoc tests were performed to determine pairwise differences. Effect sizes were calculated using eta-squared (η^2^) and are reported in the corresponding tables to provide a measure of practical significance, particularly for results approaching statistical significance. The data were analyzed using SPSS (version 26, IBM-SPSS Inc). Statistical significance was set at *P*  ≤0.05.

## Results

**FIGURE 3:**
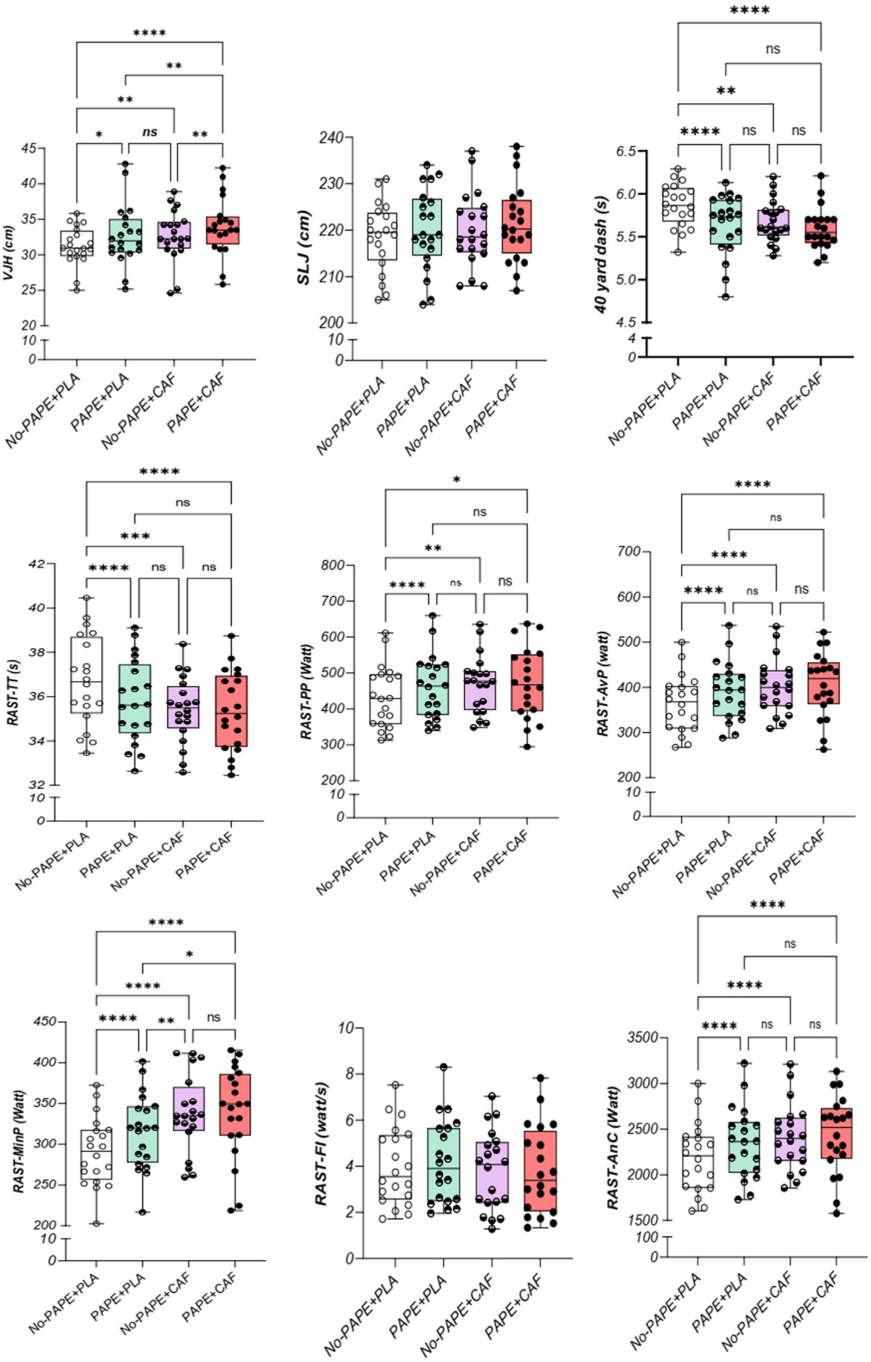
Changes in jumping, sprint, and overall performance in the running-based anaerobic sprint test (RAST) following each condition. AnC, anaerobic capacity; AvP, average power output; CAF, caffeine; FI, fatigue index; MinP, minimum power output; PAPE, postactivation performance enhancement; PLA, placebo; PP, peak power output; SLJ: standing long jump; TT, total time; VJH, vertical jump height. ns, nonsignificant; ∗*P* < 0.05, ∗∗*P* < 0.01, ∗∗∗*P* < 0.001, ∗∗∗∗*P* < 0.0001.

One-way repeated measures ANOVA showed that the main effect on VJH, 40-yard dash, RAST-TT, RAST-PP, RAST-AvP, RAST-MinP, and RAST-AnC was significant (*P* < 0.001 for all). However, no significant effect was observed on SLJ (*P* = 0.053) and RAST-FI (*P* = 0.38) (Table 1, Figure 3). The effect size values (η^2^) are reported in Table 1.

**TABLE 1:** Results of 1-way repeated measures ANOVA to determine the main effect of the interventions.

Variable	Conditions				ANOVA
	No-PAP + PLA	PAP+PLA	No-PAP + CAF	PAP + CAF	
VJH (cm)	31.2 ± 2.7	32.7 ± 4.27	32.7 ± 3.6	34.0 ± 4.1	*F* = 17.8, *P* = 0.000, η^2^ = 0.5
SLJ (cm)	218.6 ± 7.3	220.1 ± 8.7	220.0 ± 8.0	221.4 ± 8.3	*F* = 2.7, *P* = 0.053, η^2^ = 0.1
40-yard dash (s)	5.9 ± 0.2	5.6 ± 0.3	5.7 ± 0.2	5.6 ± 0.2	*F* = 12.1, *P* = 0.000, η^2^ = 0.4
RAST-TT (s)	36.7 ± 2.0	35.8 ± 1.9	35.4 ± 1.5	35.3 ± 1.8	*F* = 21.04, *P* = 0.000, η^2^ = 0.5
RAST-PP (watt)	434.1 ± 86.6	462.2 ± 88.4	470.0 ± 79.4	472.7 ± 99.2	*F* = 7.63, *P* = 0.002, η^2^ = 0.3
RAST-AvP (watt)	364.2 ± 62.6	391.4 ± 64.9	402.1 ± 61.2	406.2 ± 70.9	*F* = 20.2, *P* = 0.000, η^2^ = 0.5
RAST-MinP (watt)	290.8 ± 42.1	315.7 ± 45.9	337.3 ± 47.5	340.47 ± 56.6	*F* = 30.33, *P* = 0.000, η^2^ = 0.6
RAST-FI (watt/s)	4.0 ± 1.7	4.2 ± 1.8	3.8 ± 1.7	3.8 ± 1.9	*F* = 1.03, *P* = 0.38, η^2^ = 0.05
RAST-AnC (watt)	2185.0 ± 375.8	2348.4 ± 389.5	2412.4 ± 367.0	2437.4 ± 425.5	*F* = 20.2, *P* = 0.000, η^2^ = 0.5

Abbreviations: AnC, anaerobic capacity; ANOVA, analysis of variance; AvP, average power output; CAF, caffeine; FI, fatigue index, MinP, minimum power output; PAP, postactivation potentiation; PLA, placebo; PP, peak power output; RAST, running-based anaerobic sprint test; SLJ, standing long jump; TT, total time; VJH, vertical jump height.

The results indicated that VJH in the PAPE + CAF showed a significant enhancement when compared with the No-PAPE + PLA (*P* < 0.001), PAPE + PLA (*P* = 0.004), and No-PAPE+CAF (*P* < 0.007) conditions. Furthermore, No-PAPE + CAF also significantly improved VJH relative to No-PAPE + PLA (*P* = 0.001). Additionally, PAPE + PLA exhibited a significant increase in VJH compared with No-PAPE + PLA (*P* = 0.02). However, there was no significant difference in VJH between the PAPE + PLA and No-PAPE + CAF conditions (*P* > 0.05), as illustrated in Figure 3.

Significant enhancements in sprint performance (40-yard dash time) were observed in the PAPE + CAF (*P* < 0.001), No-PAPE + CAF (*P* = 0.002), and PAPE + PLA (*P* < 0.001) conditions as compared with No-PAPE + PLA. However, no significant differences were detected between the other pairings (*P* > 0.05) (Figure 3).

Analysis of RAST-TT demonstrated that the PAPE + CAF, PAPE + PLA, and No-PAPE + CAF conditions resulted in a significant improvement compared with the No-PAPE + PLA condition (*P* < 0.001). However, there were no significant differences in RAST-TT between the PAPE + PLA and PAPE + CAF conditions or between the PAPE + CAF and No-PAPE + CAF conditions (*P* > 0.05) (Figure 3).

A significant increase in RAST-PP was observed in the PAPE + CAF (*P* = 0.015), PAPE + PLA (*P* < 0.001), and No-PAPE + CAF (*P* = 0.002) conditions compared with the No-PAPE + PLA condition. However, no significant differences were identified in RAST-PP between the PAPE + PLA and PAPE + CAF conditions or between the PAPE + CAF and No-PAPE + CAF conditions (*P* > 0.05) (Figure 3).

RAST-AvP for the PAPE + CAF, PAPE + PLA, and No-PAPE + CAF conditions was significantly increased compared with No-PAPE + PLA (*P* < 0.001). However, no significant differences were observed in RAST-AvP between the PAPE + PLA and PAPE + CAF conditions or between the PAPE + CAF and No-PAPE + CAF conditions (P > 0.05) (Figure 3).

RAST-MinP in the PAPE + CAF condition was significantly improved compared with the No-PAPE + PLA (*P* < 0.001) and PAPE + PLA (*P* = 0.01). Furthermore, No-PAPE+CAF also exhibited a significant increase in RAST-MinP compared with No-PAPE + PLA (*P* < 0.001) and PAPE + PLA (*P* = 0.006). The PAPE + PLA condition also exhibited a significant increase in RAST-MinP compared with No-PAPE + PLA (P < 0.001). However, no significant differences were found in RAST-MinP between PAPE + CAF and No-PAPE + CAF (*P* > 0.05) (Figure 3).

RAST-AnC demonstrated a significant increase in the PAPE + CAF, PAPE + PLA, and No-PAPE + CAF conditions compared with No-PAPE + PLA (*P* < 0.001). However, no significant difference in RAST-AnC was observed between other pairs (*P* > 0.05) (Figure 3).

### Each sprint performance of RAST (RAST-ST_1–6_)

**FIGURE 4:**
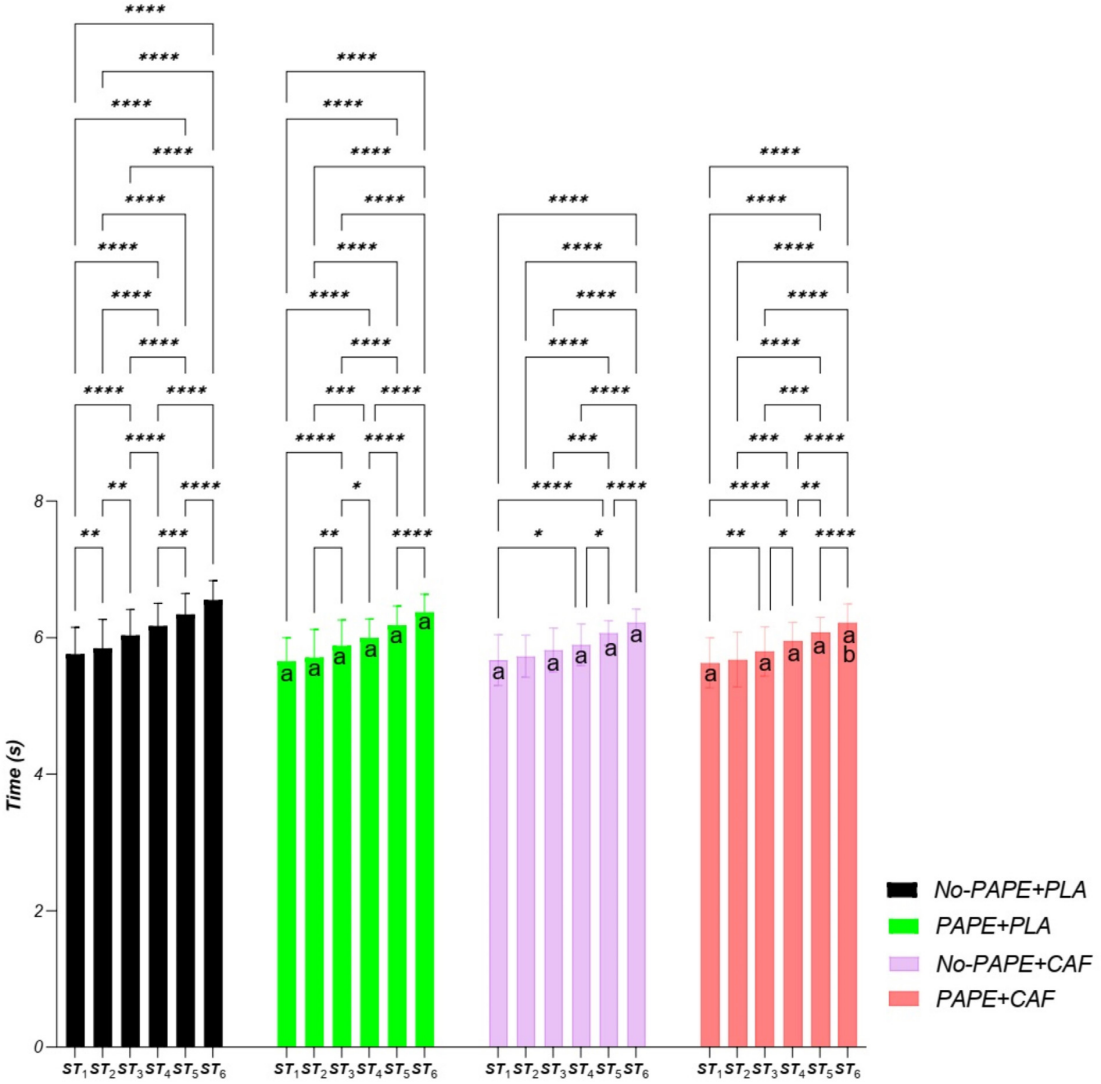
The time of each sprint (ST_1_–ST_6_) during the running-based anaerobic sprint test (RAST). (a) Significant difference with No-PAPE + PLA. (b) Significant difference with PAPE + PLA. Significant difference between pairs of sprint times: ∗*P* < 0.05, ∗∗*P* < 0.01, ∗∗∗*P* < 0.001, ∗∗∗∗*P* < 0.0001. CAF, caffeine; PAPE, postactivation performance enhancement; PLA, placebo.

Two-way repeated measures ANOVA showed that the main effect of time, condition, and interaction on RAST-ST_1–6_ was significant (Table 2, Figure 4). The effect size values (η^2^) are reported in Table 2.

**TABLE 2:** Results of 2-way repeated measures ANOVA to determine the main effect of interventions on time of each sprint in RAST (RAST-ST_1_–ST_6_).

Variable	Conditions				2-way ANOVA		
	No-PAP + PLA	PAP + PLA	No-PAP + CAF	PAP + CAF	Time	Condition	Interaction
RAST-ST_1_ (s)	5.76 ± 0.39	5.65 ± 0.34	5.67 ± 0.37	5.63 ± 0.37	*F* = 141.22 *P* = 0.000 η^2^ = 0.9	*F* = 21.4 *P* = 0.000 η^2^ = 0.5	*F* = 3.3 *P* = 0.001 η^2^ = 0.14
RAST-ST_2_ (s)	5.84 ± 0.42	5.70 ± 0.41	5.72 ± 0.31	5.67 ± 0.40			
RAST-ST_3_ (s)	6.03 ± 0.38	5.88 ± 0.38	5.82 ± 0.32	5.80 ± 0.36			
RAST-ST_4_ (s)	6.17 ± 0.33	5.99 ± 0.27	5.89 ± 0.30	5.95 ± 0.27			
RAST-ST_5_ (s)	6.34 ± 0.30	6.18 ± 0.28	6.07 ± 0.18	6.7 ± 0.21			
RAST-ST_6_ (s)	6.55 ± 0.28	6.37 ± 0.26	6.22 ± 0.19	6.22 ± 0.27			

Abbreviations: ANOVA, analysis of variance; CAF, caffeine; PAP, postactivation potentiation; PLA, placebo; RAST-ST, running-based anaerobic sprint test sprint time.

The results indicated a significant improvement in RAST-ST_1_ for the PAPE + CAF (*P* = 0.037), PAPE + PLA (*P* < 0.001), and No-PAPE + CAF (*P* = 0.046) conditions compared with No-PAPE + PLA (Figure 3). However, no significant differences in RAST-ST_1_ performance were observed between the other condition pairs (*P* > 0.05) (Figure 4).

RAST-ST_2_ was significantly improved in PAPE + PLA compared with No-PAPE + PLA (*P* < 0.001). However, no significant difference in RST-ST_2_ performance was observed between other pairs (*P* > 0.05) (Figure 4).

RAST-ST_3_ was significantly improved in PAPE + CAF (*P* < 0.001), PAPE + PLA (*P* < 0.001) and No-PAPE + CAF (*P* = 0.013) compared with No-PAPE + PLA. However, no significant differences in RST-ST_3_ performance were observed between the other condition pairs (*P* > 0.05) (Figure 4).

RAST-ST_4_ was significantly improved in PAPE + CAF (*P* = 0.004), PAPE + PLA (*P* < 0.001) and No-PAPE + CAF (*P* < 0.001) compared with No-PAPE + PLA. However, no significant differences in RST-ST_4_ performance were observed between the other condition pairs (*P* > 0.05) (Figure 4).

RAST-ST_5_ was significantly improved in PAPE + CAF (*P* < 0.001), PAPE + PLA (*P* < 0.001) and No-PAPE + CAF (*P* < 0.001) conditions compared with No-PAPE + PLA. However, no significant differences in RST-ST_5_ performance were observed between the other condition pairs (*P* > 0.05) (Figure 4).

The findings regarding RAST-ST_6_ indicated a significant enhancement in the PAPE + CAF when compared with the No-PAPE + PLA (*P* < 0.001) and the PAPE + PLA (*P* = 0.015) conditions. Additionally, both the No-PAPE + CAF and PAPE + PLA showed significant improvements in RAST-ST_6_ relative to the No-PAPE + PLA (*P* < 0.001). However, no significant differences were noted in RAST-ST_6_ performance between the other pairwise comparisons (*P* > 0.05) (Figure 4).

## Discussion

This study suggests that integrating PAPE with CAF supplementation led to significant improvements in explosive power, sprinting ability, and anaerobic capacity among recreationally active males. Notably, the PAPE + CAF group exhibited marked increases in VJH, 40-yard sprint times, and various metrics from the RAST when compared with other groups, including No-PAPE + PLA, No-PAPE + CAF, and PAPE + PLA, thereby underscoring the performance-enhancing effects of this combined approach. However, no significant effects were observed in the SLJ and RAST-FI, indicating that these measures were less sensitive to the intervention.

### Jumping performance

The study revealed significant improvements in VJH in response to the interventions, particularly in the PAPE + CAF condition, while no significant change was observed in the SLJ. This contrast between the 2 measures is intriguing, as both tests assess explosive power, but their demands differ in key ways. The VJH is primarily a measure of vertical explosive strength, emphasizing the ability to generate force rapidly against gravity [40,41]. It heavily engages the lower body, particularly the knee extensors, plantar flexors, and hip flexors, with minimal horizontal displacement [40,41]. This type of jump is directly influenced by the explosive power of the lower body, which is often targeted in plyometric training (such as SJs and bounds) [[40], [41], [42]]. Given that PAPE, particularly with CAF, enhances neuromuscular activation and potentiates muscle performance [8,12,19,28], it is not surprising that this condition led to significant improvements in VJH. The enhancement seen in the VJH, particularly in the PAPE + CAF condition, could be attributed to the combined effects of CAF-induced central nervous system activation [12,30] and the potentiation from the PAPE warm-up [8,19,21,43], which likely primed the neuromuscular system for maximal explosive output [8,9,12,18,30]. This result is consistent with prior studies suggesting that PAPE, when combined with CAF, can boost vertical jump performance by optimizing muscle recruitment and force production [7,19]. However, Rappelt et al. (2024) [6] demonstrated that in healthy young males and females, the application of PAP enhancement (PAPE) through resistance exercises, whether static or dynamic squats, did not yield a significant impact on CMJ performance when compared to a targeted muscle warm-up. It should be noted that compared with the study of Rappelt et al. [6], the present study used a plyometric protocol to induce PAPE, which can contribute to the inconsistency of the results related to VJH, especially in the PAPE + PLA condition compared with the No-PAPE + PLA condition. It is important to highlight that the plyometric exercises utilized in this study are closely related to the CMJ test employed for VJH assessment, as they exhibit comparable biomechanical characteristics and activate the same muscle groups. Additionally, Till et al. (2009) [44] found that PAPE, which incorporates both strength training and jumping exercises, does not have a significant impact on vertical jump performance. The researchers noted a significant variation in individual responses to PAPE, with changes ranging from −7.1% to +8.2% [44]. Therefore, it is crucial to consider factors such as methodology, training volume, load, recovery periods, and individual variability when implementing PAPE in practice and when planning future research investigations.

In contrast, the SLJ involves both horizontal and vertical components, requiring coordination between the lower body muscles for a combined horizontal and vertical takeoff [45,46]. The marginal nonsignificance of SLJ performance (*P* = 0.053) warrants further discussion, especially considering the small to moderate effect size (η^2^ = 0.1), indicating that there might have been subtle influences on the SLJ that were not statistically detectable. The relatively small sample size (*n* = 20) could also contribute to this outcome, as a larger sample might reveal a more pronounced effect. Additionally, it is important to consider the potential influence of individual variability in technique and execution [45,46]. The SLJ performance can be influenced by a variety of individual factors such as jumping technique, prior experience, flexibility, and neuromuscular coordination [46,47]. The differential response in VJH and SLJ can also be explained by the underlying physiological mechanisms at play. Vertical jumps primarily test the ability to overcome gravity, which is heavily influenced by muscle force production in a vertical direction [41,45,48]. This is where PAPE and CAF have been shown to have significant effects. CAF increases central nervous system activation and enhances muscle contractility [12,28,30], while PAPE increases neuromuscular efficiency and muscle recruitment [8,9], both of which contribute to improved vertical force production. Because VJH focuses on vertical force output, these mechanisms are highly aligned with the demands of the test, leading to greater improvements in performance [42]. Conversely, the SLJ involves more complex mechanics, as it requires the generation of horizontal force in addition to vertical force [45]. This demands coordination between various muscle groups, including the hip extensors, glutes, and calves, as well as a well-timed SSC [[45], [46], [47]].Although both PAPE and CAF can enhance force production and muscle recruitment, their effects might not be as pronounced in the horizontal plane, where coordination and technique are paramount. It is possible that the interventions did not sufficiently alter the SLJ technique or the ability to generate horizontal power to a degree that would produce significant changes in performance.

In summary, the study provides valuable insights into the differential effects of PAPE and CAF supplementation on vertical and horizontal jump performance. Although VJH showed significant improvement in response to the interventions, SLJ did not exhibit similar changes. This discrepancy can be attributed to the distinct demands of each test, with VJH primarily testing vertical force production, which is more directly influenced by the interventions, and SLJ involving more complex, coordinated movement that may not have been sufficiently targeted by the current training and supplementation protocols. Future studies could explore modifications to the SLJ training protocol or consider different doses and timing of interventions to better address the horizontal power demands of the test.

### Sprint performance

The present study found significant improvements in 40-yard dash performance for the PAPE + CAF, No-PAPE + CAF, and PAPE + PLA conditions when compared with the No-PAPE + PLA. These results suggest that both PAPE and CAF supplementation, either separately or combined, can enhance sprint performance in recreationally active males. However, no significant differences were observed for the other pairings, highlighting the need for a deeper examination of the individual contributions of each intervention and their potential synergistic effects. Although no previous research has directly assessed the combined effects of PAPE and CAF on 40-yard dash performance, several studies have investigated their separate effects or the synergistic impacts of other performance-enhancing interventions [7,10,[49], [50], [51]]. The present findings are supported by other studies showing that both PAPE and CAF can independently enhance performance in high-intensity, short-duration sprints [10,50,51]. For example, Matusiński et al. (2022) [51] demonstrated that PAPE effectively potentiates sprint performance. In another study, Bomfim et al. (2011) [50] showed that the drop jumps PAPE protocol increased vertical jump performance and sprint speed in high-performance athletes. This study highlighted that the effectiveness of PAPE induction is influenced not only by the protocol design but also by the timing and nature of the training used [50]. Similarly, consistent findings from previous studies indicate that CAF supplementation contributes to improvements in performance during both single and repeated sprints [10,14,49,52], which likely contributed to the sprint improvements observed in the No-PAPE + CAF. Nevertheless, some studies have failed to support the effectiveness of PAPE alone or in combination with CAF for improving sprint performance. For example, a meta-analysis conducted by Loturco et al. (2024) [53] on elite sprinters revealed that PAPE protocols did not significantly enhance sprint performance. The differences in the outcomes of the present study could be attributed to variations in the participants’ training status and fitness level (recreationally active males compared with elite sprinters). This argument is supported by the fact that training history and age are recognized as key factors influencing the PAPE response [9,54]. Notably, a meta-analysis that considered moderating factors such as training background and strength level found that PAPE leads to significant sprint performance enhancements with a moderate effect size [21]. This meta-analysis indicated that both weaker and stronger individuals can experience PAPE-related performance gains, although the timing of the induced potentiation differs between them [21]. However, conflicting findings exist, suggesting that the impact of training history requires further investigation in future research [9,54]. Although the increase in speed performance noted in this study is a new finding, it is in agreement with prior research. To further substantiate this, 2 studies have explored the interactive effects of PAPE and CAF on activities related to power, such as jumping, agility, and kick speed, highlighting their synergistic role in boosting speed and power performance [7,19].

The significant improvements in sprint performance observed in the PAPE + CAF, No-PAPE + CAF, and PAPE + PLA conditions can be explained by the mechanisms underlying PAPE and CAF separately and how they may interact. PAPE is known to enhance the force output of muscles by priming the neuromuscular system [8,9,21], resulting in improved sprint performance [21]. The mechanism is believed to be linked to the potentiation of muscle fibers, especially type II fibers, which are responsible for generating high-intensity power [8,9,21,43]. This increased muscle readiness likely resulted in faster sprint times, as evidenced by the significant improvements observed in the current study. CAF, however, is widely recognized as an ergogenic aid that enhances performance in high-intensity exercise [10,11,14,55]. It works by blocking adenosine receptors in the brain, thereby increasing the release of dopamine and adrenaline [12,28,30,32]. This leads to greater alertness, reduced perception of effort, and a delayed onset of fatigue, all of which contribute to improved sprinting performance [12]. In the present study, CAF supplementation likely contributed to the performance gains observed in the No-PAPE + CAF condition, aligning with previous findings that suggest CAF is a potent stimulant for improving sprinting ability [55]. Although the combination of PAPE and CAF resulted in the greatest improvement in sprint performance, the lack of a statistically significant difference between PAPE + CAF and the individual conditions (PAPE + PLA and No-PAPE + CAF) suggests that their combined effect may not be truly synergistic in enhancing 40-yard dash performance. Although mean sprint times improved more in the PAPE + CAF condition, the absence of a significant difference indicates that the benefits of PAPE and CAF might not be additive in this context. This contrasts with previous findings showing a clear synergistic effect on agility and kick speed [7]. One possible explanation is that sprinting relies more heavily on maximal acceleration and stride mechanics, which may not be equally influenced by both interventions when combined [55]. Additionally, individual variability in responsiveness to PAPE and CAF may have contributed to the observed results, potentially diluting any additive effects. Future studies should further investigate the interaction between these interventions in sprint-specific tasks, considering factors such as timing, dose-response relationships, and participant training status.

The results of the present study highlight the efficacy of both PAPE and CAF supplementation as independent performance enhancers for sprinting ability. The significant improvements observed in the PAPE + CAF, No-PAPE + CAF, and PAPE + PLA conditions underscore the potential of these interventions for enhancing short-duration, high-intensity activities such as the 40-yard dash. Although the combination of PAPE and CAF showed the most pronounced benefits, CAF alone also proved to be an effective intervention for improving sprint performance, aligning with findings from previous research on the ergogenic effects of CAF [10,12,55]. However, the lack of significant differences between certain pairings suggests that further research is needed to identify the specific contexts in which these interventions can have the greatest impact. Understanding the mechanisms behind the effects of PAPE and CAF, as well as the interactions between these factors, will be crucial for developing more targeted performance-enhancing strategies in the future.

### RAST performance parameters

The results of this study demonstrated that the combination of PAPE and CAF supplementation significantly improved various RAST parameters in recreationally active males. Significant enhancements were observed in RAST-TT, RAST-PP, RAST-AvP, RAST-MinP, and RAST-AnC in the PAPE + CAF, PAPE + PLA, and No-PAPE + CAF groups compared to the No-PAPE + PLA group (*P* < 0.001). Improvements in individual sprint performance (RAST-ST_1–6_) were particularly notable in the PAPE + CAF, PAPE + PLA, and No-PAPE + CAF conditions. These results underscore the beneficial effects of combining PAPE and CAF supplementation on anaerobic capacity. Moreover, the RAST-FI showed no significant improvement (*P* = 0.38, η^2^ = 0.05), which could be due to the specific nature of the fatigue measure. PAPE and CAF primarily enhance explosive power and short-duration sprint performance rather than fatigue resistance over prolonged high-intensity efforts. Therefore, the interventions might have been more effective in the initial phases of performance (e.g., vertical jumps, sprint times) but did not significantly impact the endurance aspects of fatigue, which depend on anaerobic endurance and sustained performance. Consequently, RAST-FI results suggest that although the interventions showed potential for improving certain performance measures, their effects on endurance and more technique-sensitive movements may require further investigation. However, it is important to recognize that the performance improvements across the sprint series (RAST-ST_1–6_) in the PAPE, CAF, and PAPE + CAF conditions do suggest that recovery was somewhat facilitated during repeated sprints.

Consistent with these findings, some previous studies have shown that CAF [10,56,57] or PAPE [58,59] can independently lead to improvements in repeated sprint tests. This may be a result of improved recovery between sprints rather than an outright enhancement in fatigue resistance, aligning with the idea that PAPE and CAF primarily support power output in the short term and recovery during intense bursts of activity. The facilitatory effects of PAPE on the neuromuscular system, combined with the analgesic mechanisms of CAF, provide a strong physiological basis for the observed improvements in RAST performance. PAPE enhances neuromuscular efficiency by increasing motor unit recruitment and excitation-contraction coupling, leading to greater power output and sprint performance [8,9,21,60]. This aligns with the observed increases in RAST-PP, RAST-AvP, RAST-MinP, RAST-TT, RAST-AnC, and RAST-ST_1–6_, as potentiated muscles can generate force more rapidly and sustain higher intensities across repeated efforts [58,59]. Additionally, CAF’s analgesic effects, mediated by adenosine receptor antagonism [12,30,52], may reduce perceived exertion and muscle discomfort [12,33], allowing athletes to maintain optimal sprint performance throughout the test. Although fatigue resistance, as measured by RAST-FI, did not show significant changes, the improved sprint times suggest that PAPE and CAF may have contributed to better power maintenance across sprints, indirectly facilitating recovery between efforts. Furthermore, the higher RAST-MinP values observed in the PAPE + PLA, No-PAPE + CAF, and PAPE + CAF conditions compared with No-PAPE + PLA indicate that these interventions not only enhanced RAST-PP and RAST-AvP but also likely facilitated recovery between sprint repetitions, contributing to sustained power output in the later stages of the test. These findings suggest that PAPE and CAF may improve power stability during RAST by enhancing motor unit recruitment and reducing perceived fatigue. Recent research further supports the synergistic effects of PAPE and CAF on anaerobic performance [7,19]. Zhang et al. (2024) [7] demonstrated that a PAPE protocol involving a 10-s all-out cycling sprint, when combined with 3 mg CAF/kg intake, significantly enhanced PP and AvP during the Wingate Anaerobic Test in highly trained boxers. Notably, CAF amplified the effects of PAPE on PP and participants’ subjective perception of power [7], suggesting an additional ergogenic benefit. These findings align with the present study, in which the PAPE + CAF condition led to superior power output (RAST-PP, RAST-AvP, RAST-AnC) and sprint performance (RAST-ST_1–6_) improvements in the RAST test. The increase in postexercise blood lactate concentration observed by Zhang et al. (2024) [7] also suggests a greater reliance on anaerobic metabolism, which may explain the enhanced RAST-AnC seen in the present study. Collectively, these findings reinforce the role of PAPE and CAF as effective strategies for optimizing anaerobic capacity and repeated-sprint performance, particularly in high-intensity intermittent sports and repeated sprints.

In summary, the combination of PAPE and CAF supplementation effectively enhanced key anaerobic performance measures in the RAST test, particularly in terms of power output and sprint performance across repeated efforts. Although no significant changes were observed in fatigue resistance, the observed improvements in RAST-ST_1–6_ suggest that these interventions may have facilitated recovery between sprints. These findings highlight the potential of PAPE and CAF to optimize explosive performance, making them valuable strategies for athletes engaged in high-intensity, repeated-sprint activities.

This study has several limitations that should be acknowledged. The small sample size of 20 recreationally active male participants limits the generalizability of the findings, particularly to female or elite athletic populations. The use of a specific plyometric-based PAPE protocol may not fully represent other PAPE strategies, such as resistance training or sport-specific conditioning, reducing the applicability of the results to various contexts. Furthermore, a fixed dosage of 6 mg CAF/kg BM was used, without accounting for individual variability in CAF sensitivity or metabolism. The study focused solely on acute performance enhancements, leaving long-term effects unexplored. Performance outcomes were limited to explosive and anaerobic metrics, excluding other relevant aspects such as endurance or recovery. Although sessions were conducted at a consistent time, the fixed timing of CAF ingestion and PAPE protocols may not align optimally with all individuals’ performance peaks. Additionally, no significant effects were observed on the FI, raising questions about the interventions’ role in fatigue resistance. Lastly, strict dietary controls, including CAF washout periods, may not reflect real-world scenarios, potentially limiting the ecological validity of the findings. These limitations underscore the need for further research with diverse populations, varied protocols, and extended performance metrics to fully elucidate the potential of combining PAPE with CAF supplementation.

### Conclusion

This study demonstrates that combining PAPE with CAF supplementation significantly enhances explosive power, sprint performance, and anaerobic capacity in recreationally active males. The synergistic effects observed in VJH, sprint times, and RAST metrics highlight the practical potential of this approach for high-intensity activities. Although further research is needed to explore long-term effects, optimal dosages, and broader applicability, these findings offer valuable guidance for improving athletic performance through combined nutritional and conditioning strategies.

## Author contributions

The authors’ responsibilities were as follows – JN, AN: conceptualization; JN, MH, AN: methodology; AN, JN, MH, FE: software; AN, ARD, NH, MS: formal analysis; JN, AN, FE: investigation; NH, MS, ARD: resources; NH, MS, AN, FE: data curation; AN: wrote the original draft; ARD, JN, MS: reviewed and edited the manuscript; AN, JN, MH: supervision; JN, AN: project administration; JN, AN, FE: funding acquisition; and all authors: read and approved the final manuscript.

## Data availability

Data described in the manuscript will be made available upon request.

## Funding

The authors reported no funding received for this study.

## Conflict of interest

The authors report no conflicts of interest.
